# Unary Non-Structural Fertilizer Response Model for Rice Crops and Its Field Experimental Verification

**DOI:** 10.1038/s41598-018-21163-w

**Published:** 2018-02-12

**Authors:** Mingqing Zhang, Juan Li, Fang Chen, Qingbo Kong

**Affiliations:** 1 Soil and Fertilizer Institute, Fujian Academy of Agricultural Science, Fuzhou, 350013 China; 2 Key Laboratory of Aquatic Botany and Watershed Ecology, Wuhan Botanical Garden, Chinese Academy of Sciences, Wuhan, 430074 China; 3 Wuhan Office of International Plant Nutrition Institute, Wuhan, 430074 China

## Abstract

The quadratic polynomial fertilizer response model (QPFM) is the primary method for implementing quantitative fertilization in crop production, but the success rate of this model’s recommended fertilization rates in China is low because the model contains a high setting bias. This paper discusses a new modelling method for expanding the applicability of QPFM. The results of field experiments with 8 levels of N, P, or K fertilization showed that the dynamic trend between rice yield increases and fertilizer application rate exhibited a typical exponential relationship. Therefore, we propose a unary non-structural fertilizer response model (NSFM). The responses of 18 rice field experiments to N, P, or K fertilization indicated that the new models could significantly predict rice yields, while two experimental fitting results using the unary QPFM did not pass statistical significance tests. The residual standard deviations of 13 new models were significantly lower than that of the unary QPFM. The linear correlation coefficient of the recommended application rates between the new model and the unary QPFM reached a significant level. Theoretical analysis showed that the unary QPFM was a simplified version of the new model, and it had a higher fitting precision and better applicability.

## Introduction

At present, fertilizer response models can be divided into mechanistic and experiential models^1^, and between them, with “semi-mechanistic and semi-experiential” qualities, is the so-called non-structural fertilizer response model^2^. Due to the complex structure and parameters of the mechanistic model, it remains difficult to popularize. The experiential model, conversely, is one of the main approaches used to recommend fertilization rates due to the following reasons: In experiential models, the unary quadratic polynomial model can better reflect the quantitative relationship between crop yield and fertilizer application rate. Additionally, it is relatively simple and easy to calculate and estimate the model’s parameters. Finally, because binary or tertiary quadratic polynomial models were set up on the basis of the unary model, these have been widely used to recommend fertilization rates for crops^3–9^.

However, in fertilization practice, because of the complexity of agricultural conditions, and because unary or multiple quadratic polynomial models have already been established according to field experimental results, the equation effect curve or the shape of the surface varies greatly^10,11^. In experiential models, if the established model can satisfy the following conditions^12^, then it conforms to the general fertilizer efficiency rule of plant nutrition: (1) the algebraic sign of the monomial coefficient is positive; (2) the algebraic sign of the quadratic coefficient is negative; (3) the model has a maximum output point which falls within the output range of crop planting experiments; and (4) the maximum fertilization rate and economic fertilization rate estimated by the marginal product derivative method are within the scope of the fertilization design. The quadratic polynomial fertilizer response model is used as the typical fertilizer response model. On the other hand, if there is a condition that cannot be satisfied, the model is called a non-typical fertilizer response model. Many studies have shown that the accuracies of the typical unary, binary and tertiary quadratic polynomial fertilizer response models are only approximately 60%, 40.2%^10,11^, and 23.6%^12^, respectively. Many non-typical models appeared during model establishment, which severely weakened the accuracy of computation and the practical value of quadratic polynomial models. To this end, domestic and international researchers studied model selection and applicability, experimental design and parameter estimation; also, class feature effectiveness modelling and the non-typical model based on the recommended fertilization optimization method were studied. These models formed the bases for suggesting improvement measures^2^, which have not led to a satisfactory solution to the related problem to date. Conversely, improvements to the model itself were rarely reported.

Paddy rice is the most important food crop in China. In this paper, a single factor N, P, or K fertilizer efficiency experiment forms the basis for discussing the limitations of the unary quadratic polynomial fertilizer response model and the improved method. Next, we developed a unary non-structural fertilizer response model in order to improve the fitting precision of fertilizer effect models and expand their applicability.

## Results

### Rice yield response to unit nutrient application and model improvement

The mathematical expression of a common quadratic polynomial with one variable is

1 $${\rm{Y}}={b}_{0}+{b}_{1}{\rm{X}}+{b}_{2}{{\rm{X}}}^{2}$$

where X is the application rate of fertilizer and Y is the crop yield. Its differential expression is

2 $${\rm{d}}{\rm{Y}}/{\rm{d}}{\rm{X}}={b}_{1}+2{b}_{2}{\rm{X}}$$

The result shows that the quantitative relationship between crop yield increase per unit nutrition and its application rate is assumed to be linear in the unary quadratic polynomial fertilizer response model.

To examine the rationality of the linear assumption of the model (2), the rice yield response to a per unit nutrient application was explored in field experiments in Datian county and Nanan City in Fujian province using 8 fertilization rates. The application rates of N, P_2_O_5_, K_2_O in three field experiments and their yields are shown in Table 1.

**Table 1:** Effect of N, P and K fertilizer application rates on rice yields.

Sites	Nutrient	Items	Application rate of fertilizer and the rice yield							
Datian county	N	X(kg/hm^2^)	0	37.5	75.0	112.5	150.0	187.5	225.0	262.5
		Y(kg/hm^2^)	5051 ± 123	5801 ± 102	6483 ± 121	6834 ± 68	7001 ± 173	6900 ± 252	6675 ± 93	6600 ± 33
Nan’an city	P_2_O_5_	X(kg/hm^2^)	0	22.5	45.0	67.5	90.0	112.5	135.0	157.5
		Y(kg/hm^2^)	6809 ± 286	7274 ± 191	7490 ± 180	7616 ± 219	7577 ± 344	7449 ± 260	7236 ± 180	6953 ± 125
Datian county	K_2_O	X(kg/hm^2^)	0	30	60	90	120	150	180	210
		Y(kg/hm^2^)	7670 ± 87	8570 ± 321	9215 ± 379	9485 ± 115	9225 ± 189	8763 ± 76	8411 ± 100	7880 ± 275

Notes: X denotes application rate; Y denotes rice yield (mean ± SD).

**Figure 1:**
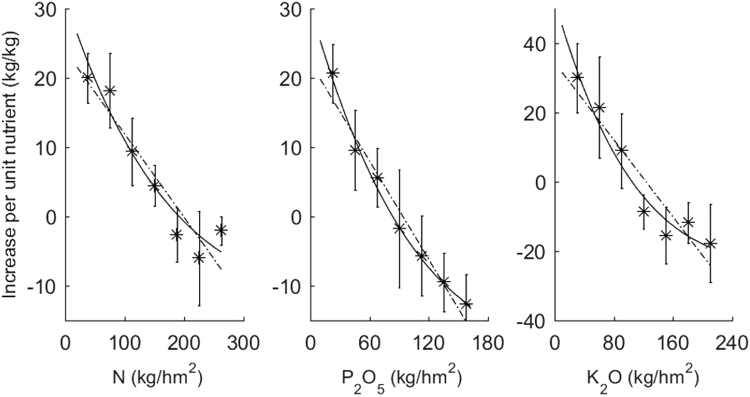
Rice yield response to application rate of per kg N, P_2_O_5_ and K_2_O. The solid line shows the fitted results of the exponential model; the dashed line showed the fitted results of the linear model.

First, we calculated the rice yield increase in response to unit nutrition in the treatments with different fertilization rates as d*Y*/d*X* = Δ*Y*/Δ*X* = (Y_*i*+1_ − Y_*i*_)/(X_*i*+1_ − X_*i*_), where *i* is the fertilization serial number (i.e., *i* = 1, 2, 3…, 7). Next, a two-dimensional chart (Fig. 1) based on the value of ΔY/ΔX and the application rate was drawn. The results indicate that the increase in rice yield drops rapidly with the increase of N, P and K fertilizer application rates in earlier stages, which are almost linearly related. However, with a further increase in fertilization rates, the decline gradually slows. As a whole, the increase of rice yield per unit nutrition decreases exponentially with the increase of fertilization rates.

The dynamic characteristics of the increasing rate per unit nutrition in Fig. 1 suggest that the linear hypothesis of the model (2) should be modified. To obtain a simplified and improved model, we set the unit area soil nutrient supply equivalent to *s*_0_, which is roughly assumed to be a constant, and soil nutrient supply *s*_0_ and fertilization rate may be additive. The soil nutrient supply capacity is thus described as x = *s*_0_ + X, which includes chemical fertilizer X applied to soil. On the basis of the principles of calculus, dx = d(*s*_0_ + X) = dX, and dY/dx = dY/d(*s*_0_ + X) = dY/dX. Ordered as 2*b*_2_/*b*_1_ = −*c*, *b*_1_ = *a*, the model could be translated as dY/dX = *a*[1 − *c*(*s*_0_ + X)], where *c* describes the effect of fertilization on yield. Due to the relationship between yield and increasing fertilization having both a linear and an exponential effect (Fig. 1), it could be further modified to dY/dX = *a*[1 − *c*(*s*_0_ + X)] *e*^−*c*(*s*0+X)^. To make *A* = *a*e^−*cs*0^, we can obtain an improved model (3):

3 $$\frac{{\rm{d}}Y}{{\rm{d}}{\rm{X}}}=A[1-c({{\rm{s}}}_{0}+{\rm{X}})]{e}^{-cX}$$

According to the experimental results in Table 1, regression modelling was carried out using model (3). Figure 1 shows that the fit of the exponential model (solid line) is clearly superior to that of the linear model (dashed line). The quantitative comparison in Table 2 shows that the results of eight fertilization rates can be significantly predicted by both the linear model (2) and the exponential model (3). However, the statistically significant index of *F* values and the goodness of fit *R*^2^ values of the exponential model are both relatively larger than the linear model. Additionally, the standard deviation of fit residuals that are used to evaluate the regression model fitting effect^13^, were significantly lower for the exponential model (3) than for the linear model (2). Therefore, the exponential model (3) has a higher fitting precision and could better describe the relationship between the increase in production of per unit nutrition and the application rate of fertilizers.

**Table 2:** Comparison of fitting results of the exponential model and linear model of rice yield response to unit nutrition.

Sites	Nutrients	Model (3)						Model (2)				
		Model coefficient			Statistical index			Model coefficient		Statistical index		
		*A*	*c* × 10^3^	*s*_0_	*F*	*R* ^2^	*S*	*a*	*b*	*F*	*R* ^2^	*S*
Datian county	N	52.81	3.134	131.020	26.64**	0.930	2.994	23.966	−0.1204	40.28**	0.890	3.765
Nan’an city	P_2_O_5_	74.23	4.887	120.610	299.54**	0.993	1.048	22.229	−0.2368	119.55**	0.960	2.579
Datian county	K_2_O	102.40	4.864	99.362	41.38**	0.954	4.503	34.429	−0.2786	40.07**	0.889	6.891

Note: The linear model (2) was rewritten as y = *a* + *b*X. S is the standard deviation of fitting residuals; *R*^2^ is goodness of fit, “**” means level of significance (*p* < 0.01).

### Unary non-structural fertilizer response model and its verification

To integrate the model (3), the integration model is Y = *A*(*s*_0_ + X) *e*^−*c*X^ + *C*, where *C* is the integral constant. When both the soil nutrient supply equivalent *s*_0_ and the fertilization rate X are equal to zero, the crop yield must be zero; thus, *C* is zero. The unary non-structural fertilizer response model is

4 $${\rm{Y}}=A({s}_{0}+{\rm{X}}){e}^{-{\rm{cX}}}$$

where Y is the rice yield, X is the fertilization rate, *s*_0_ is the soil nutrient supply equivalent and *c* is the coefficient of increased yield response to nutrient. *A* = *ae*^−*cs*0^ is the conversion coefficient between soil fertility and rice yield when X = 0, and reflects soil productivity.

**Figure 2:**
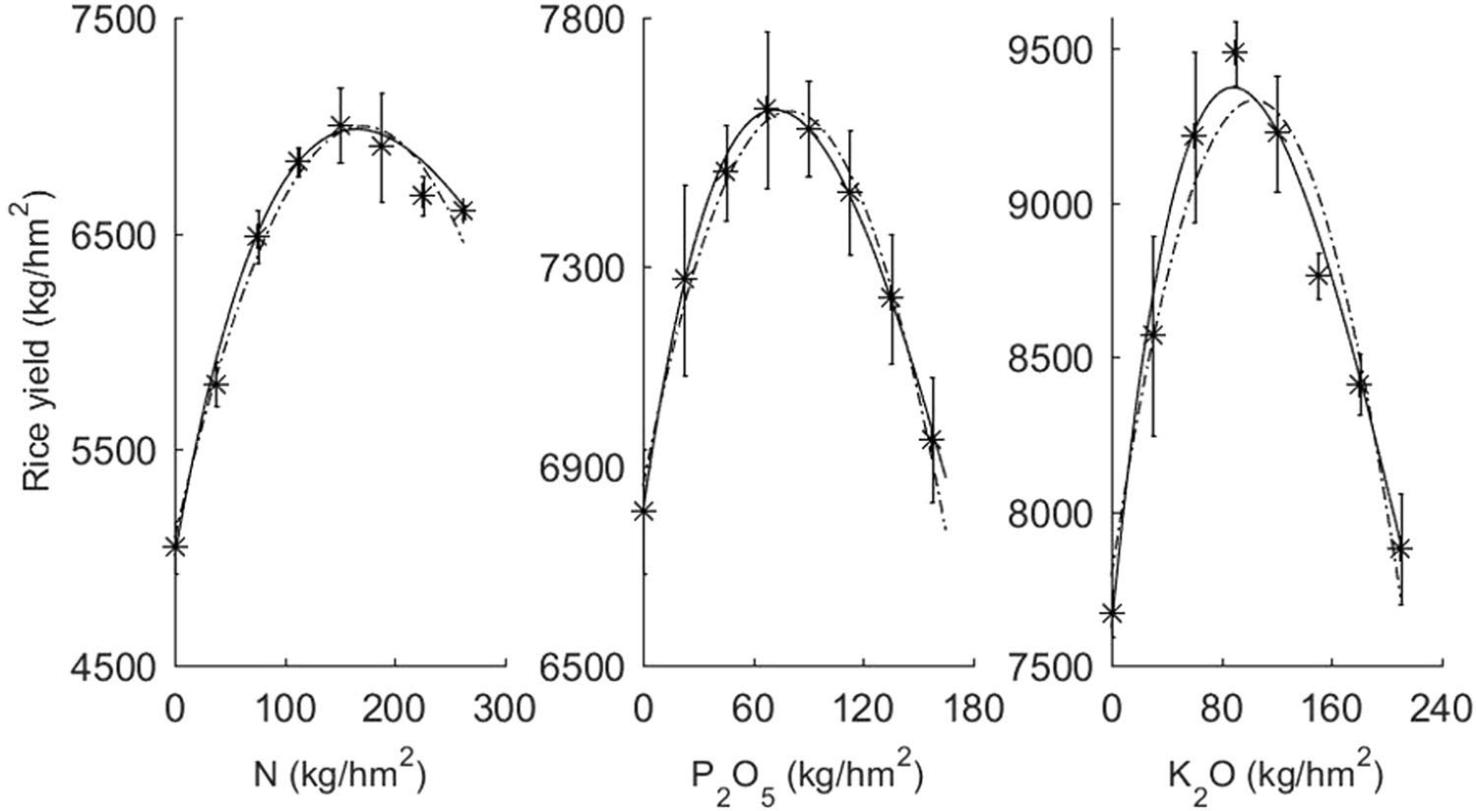
Comparisons of fitting results between model (4) and model (1). The solid line is the fitted results of non-structural fertilizer response models, and the dashed line is the fitted results of the quadratic polynomial model.

The solid and dotted lines of Fig. 2 are represented by model (4) and model (1). According to the results of field experiments, two kinds of fertilizer response models can better fit the results of eight fertilizer application rates of nitrogen, phosphorus and potassium on paddy rice.

The fit comparison of model (4) and model (1) based on 18 field experiments is shown in Table 3. The results showed that both the statistically significant index of *F* value and the goodness of fit *R*^2^ values of the unary non-structural fertilizer response model were larger and significant, while the fitting results of two of the 18 experiments were not significant with respect to the unitary quadratic polynomial models. The evaluation using index *S* of model fitting also showed that except for five field experiments (including three N fertilization, one P fertilization and one K fertilization), the residual standard deviations of 13 unary non-structural fertilizer response models were lower than those of the quadratic polynomial fertilizer response models, which shows that the fitting precision of model (4) reached 72.2% and was better than that of model (1).

**Table 3:** Comparison of fitting results between unary non-structured fertilizer response model and unary quadratic polynomial fertilizer response model.

Experiments	No.	Nutri-ents	Model (4)						Model (1)					
			Model coefficient			Statistical index			Model coefficient			Statistical index		
			*A*	*c* × 10^3^	*s*_0_	*F*	*R* ^2^	*S*	*b*_0_	*b*_1_	*b*_2_	*F*	*R* ^2^	*S*
8 fertilization rates	1	N	47.596	3.740	105.120	255.6**	0.990	77.5	5087.6	22.273	−0.065	126.7**	0.981	109.5
	2	N	75.323	5.517	40.359	12.8*	0.837	577.1	3086.6	44.886	−0.145	46.4**	0.949	323.1
	3	N	70.609	5.699	86.106	253.5**	0.990	53.5	6154.7	27.199	−0.140	60.4**	0.960	107. 9
	4	P	63.739	5.607	106.790	1189.7**	0.998	15.9	6858.5	18.775	−0.117	138.8**	0.982	46.1
	5	P	67.972	5.189	112.790	23.8**	0.905	127.7	7715.3	21.763	−0.124	21.7**	0.897	133.1
	6	P	103.370	7.362	71.182	64.9**	0.963	121.9	7587.4	29.559	−0.197	14.7**	0.855	241.0
	7	K	90.851	5.824	83.871	159.7**	0.985	30.3	7788.7	29.707	−0.143	38.8**	0.940	189.9
	8	K	57.217	4.921	104.670	17.0**	0.872	179.8	6096.2	18.697	−0.083	9.6*	0.793	228.3
4 ~ 7 fertilization rates	9	N	67.582	4.192	126.720	26.3**	0.946	197.6	8624.6	22.802	−0.087	374.5**	0.996	53.7
	10	N	77.037	5.988	63.948	16.1*	0.915	302.6	4988.8	33.911	−0.140	255.5**	0.994	79.2
	11	N	51.525	4.995	93.094	34.7*	0.972	119.4	4825.1	21.924	−0.095	31.8*	0.969	124.6
	12	N	97.207	4.710	81.760	69.4*	0.986	214.2	8097.9	42.347	−0.1427	14.8	0.937	452.0
	13	P	65.931	4.634	155.330	126.3**	0.988	26.6	10252	15.854	−0.124	280.5**	0.995	17.9
	14	K	32.950	4.185	178.880	19.3**	0.906	60.0	5920.3	8.1339	−0.043	11.0*	0.846	76.9
	15	K	46.920	3.887	164.070	30.2**	0.938	82.1	7731.3	13.863	−0.066	20.2**	0.910	98.8
	16	K	77.715	4.429	109.860	272.3**	0.995	68.6	8640.9	29.051	−0.111	32.7**	0.956	194.2
	17	K	39.449	4.173	136.380	145.7**	0.993	35.4	5394.9	13.953	−0.063	337.2**	0.997	23.3
	18	K	74.698	4.297	135.560	396.2*	0.999	39.8	10165.0	22.271	−0.098	15.5	0.938	198.2

### Recommended fertilization rates by non-structural fertilizer response model

There is a peak of rice yield within the range of fertilization rates in the unary non-structural model as shown in Fig. 2, which corresponds to the maximum yield application rate. According to the general principle of fertilizer efficiency, when the marginal yield is equal to the price reciprocal proportion of rice and fertilizer, the fertilization rate used is the fertilization rate for economic yield. Therefore, we can assign model (5) for calculating the maximum fertilization rate and model (6) for calculating the economic fertilization rate.

5 $${{\rm{X}}}_{max}=\frac{1}{c}-{s}_{0}$$

6 $${{\rm{X}}}_{{\rm{e}}{\rm{c}}{\rm{o}}}=\frac{1}{c+\frac{\beta }{{\rm{Y}}{\rm{e}}{\rm{c}}{\rm{o}}}}-{s}_{0}$$

In model (6), *β* = Px/Py, Px and Py are the market prices for unit nutrients and agricultural products, respectively. Y_eco_ is the economic yield, but it is unknown before the economic fertilization rate has been obtained. Experience indicates that the difference between maximum yield and economic yield is ordinarily very small, based on the calculated result of fertilizer response models. Therefore, Yec_o_ could be replaced by Y_max_ which is obtained from model (5). This value could also be refined using an iterative algorithm approach. Generally, approximately 3–5 iterative calculations are sufficient.

**Figure 3:**
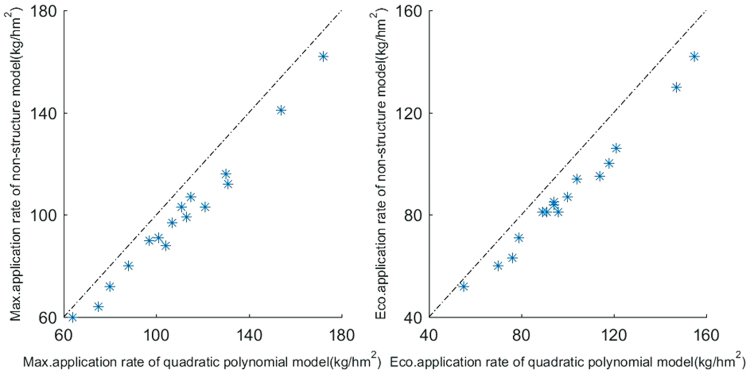
Comparison of recommended fertilization rates between the unary non-structural fertilizer response model and the unary quadratic polynomial fertilizer response model.

Based on 16 field trials (in Table 3) that passed significance tests using the unary quadratic polynomial model, the recommend fertilization rates of N, P_2_O_5_ and K_2_O were calculated by model (1) and model (4). The results showed that there is a significant linear positive correlation between recommended fertilization rates of model (1) and (4), with a correlation coefficient of 0.9868** (n = 16) for the maximum fertilization rate, and a correlation coefficient of 0.9910** (n = 16) for the economic fertilization rate. The points in Fig. 3 are distributed at the lower right of the diagonal, regardless of whether the maximum rate or economic rate is plotted. The recommended maximum fertilization rates of N, P_2_O_5_ and K_2_O using the unary non-structural fertilizer effect model were only 91.5%, 89.4% and 89.0%, respectively, of that recommended by the unary quadratic polynomial model, and the recommended economic fertilization rates were only 90.4%, 88.9% and 89.0%, respectively, of that recommended by the unary quadratic polynomial model.

### Correlation between s0 and rice nutrient absorption

**Figure 4:**
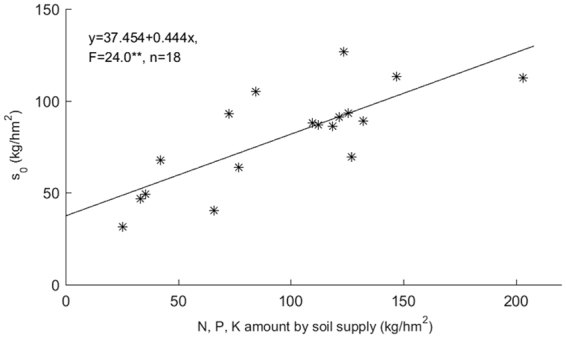
Correlation between soil nutrient-supplying amounts of N, P, K and *s*_0_.

To evaluate the reliability of the predicted values of nutrient uptake, a monadic linear regression analysis using predicted values and the measured values is common for the study of the root nutrient absorption mechanism model^14^. In this paper, the accuracy of the estimated value *s*_0_ in the non-structural fertilizer effect model was inspected using the same method. Using the CK (no fertilization) treatment, the harvested rice and rice straw yields, and the N, P and K contents in their samples, the total nutrition uptake rates of N, P and K were calculated, and the values were assumed to be the values of the indigenous soil nutrient supply during the rice production season. The estimated soil P_2_O_5_ and K_2_O values of *s*_0_ in Table 3 was converted into the equivalent P and K nutrient supply, then mapped in Fig. 4. Regression analysis showed that the relationship between the estimated value *s*_0_ (y) and the uptake of N, P and K nutrients in the CK treatment was satisfied with the linear regression model (F = 24.0**, n = 18), which indicates that there is a significant linear positive correlation between these values, and the estimated value *s*_0_ in model (4) accurately reflected the soil nutrient-supplying potential.

## Discussion and Conclusions

### Model applicability on the functional setting of yield increase per unit nutrition

The results in Fig. 1 show that a quantitative relationship between rice yield increase per unit N, P and K fertilization and their application rates was a typical exponential function, and the distribution trend of the points clearly deviates from a linear relationship. Although both the linear and the exponential models could pass statistical significance testing, the standard deviation of the residuals of the exponential model was significantly lower than that of the linear model and had a higher fitting precision.

In the fertilizer response function, based on different assumptions of the functional relationships with yield increase per unit nutrient and application rate, various fertilizer response models with different mathematical forms and applicability could be obtained. The famous Michaelis-Menten fertilizer response equation assumes that the yield increase per unit nutrient is proportional to the difference between the maximum yield and its practical output^15^. Spelman’s fertilizer response equation assumes that the ratio of yield increase per corresponding unit nutrient is constant^15^. Both of the fertilizer response models with the two assumptions can only reflect fertilization effects before reaching the maximum yield and cannot describe the yield reduction from over-fertilization. The common unary quadratic polynomial model assumes that the yield increase per unit nutrient is positively related to the difference between the maximum yield application rate and the practical fertilization rate^16^. This linear hypothesis shows that the yield increase per unit nutrient decreases linearly with the increase in fertilization before the point of maximum yield. This increase also decreases linearly with a continually symmetrical increase in fertilization after reaching the point of maximum yield. The advantages of this hypothesis are that it is simple and practical, and the unary quadratic polynomial model can result in yield reductions caused by over-fertilization; this has been verified by large fertilizer experiments globally, especially with N fertilization experiments^7,15–17^.

However, with the improvements in agricultural technology and the large increases in crop yield, especially the widespread use of new varieties with high yields and fertilizer tolerance, crop responses to fertilizers have changed dramatically^18,19^. Especially with excessive fertilization, the reduction in output has been greatly alleviated by fertilizer tolerance and the nutrient buffering capacity of soil, which causes yield increases per unit nutrition to decline with an exponential trend. Thus, the improved model (4) better reflects current agricultural production practices.

### Reliability of the unary non-structured fertilizer response model

To overcome the defects of the quadratic polynomial fertilizer response model, the unary non-structured fertilizer effect model (model (4)) was proposed in this paper. The model is obviously different in mathematical form from that of the experiential and mechanistic models of root nutrient uptake^14^, but the parameters of the model are well-defined and have a relatively simple application. For reference, fields such as population ecology name different types of models^20^; model (4) has characteristics between the mechanistic and the empirical models and is called the unary non-structural fertilizer response model. The simulation results from 18 field experiments using N, P and K fertilizers show that it has a higher fitting accuracy than the unary quadratic polynomial fertilizer response model.

*A*, *c*, and *s*_0_ parameter estimates of model (3) and model (4) did not agree with each other according to the experimental results of Table 3. The main reason is that the fertilization rate (X) and output (Y) have no uncertain relationships and the output (Y) is a random variable. ΔY in model (3), which is affected by the random wave characteristics of output (Y), was more serious than that of Y itself in model (4), which led to different parameter estimates. Clearly, random effects in model (4) were smaller than those in model (3) and the reliability of the coefficient estimate values were higher.

The data in Table 4 show the results from nine field experiments on N and P fertilization of winter wheat in northern China, conducted by Li R G^15^ and Chen L S^21^ in the 1980s. Although all of the fertilization rates and yield levels were lower than current rates, their use for verifying the unary quadratic polynomial fertilizer response model was valid. The modelling results in Table 5 show that the unary non-structured fertilizer response model was a good fit, and all nine experimental results passed statistical significance testing. The results from the second experimental site did not reach a statistically significant level using the unary quadratic polynomial fertilizer response model, but the results passed the significance test when using the unary non-structured fertilizer response model. Maximum application rate and economic application rate of the nine non-structured fertilizer response models in Table 5 were calculated using model (5) and model (6), respectively. The calculations showed that the maximum application rate and the economic application rate of model (4) and model (1) had significant linear positive correlations, with correlation coefficients of 0.979** and 0.982**, respectively. Therefore, the fertilization rate recommended based on the unary non-structured fertilizer response model should be reliable for winter wheat, as well.

**Table 4:** Effect of different application rates of N, P, K fertilizers on winter wheat yield.

No.	Fertilizer	Application rate and trial yield (kg/hm^2^)								Data source
1	N	0 (4133)	45 (5693)	90 (6458)	135 (7350)	180 (7178)	225 (6480)	—	—	References 15^th^, p 8
2	N	0 (4313)	45 (4950)	90 (5063)	135 (4875)	180 (4688)	—	—	—	References 15^th^, P 9
3	N	0 (2325)	60 (2700)	120 (3720)	180 (4260)	240 (4530)	300 (4343)	—	—	References 15^th^, P 74
4	Urea	0 (4080)	150 (4785)	300 (5355)	450 (5783)	600 (6165)	750 (5895)	—	—	References 15^th^, P 139
5	N	0 (2177)	34 (3413)	68 (4112)	101 (4514)	135 (4716)	—	—	—	References 21^th^, P 66
6	N	0 (3995)	34 (4355)	68 (4602)	101 (4658)	135 (4703)	—	—	—	References 21^th^, P66
7	N	0 (2453)	34 (3075)	68 (3585)	101 (3656)	135 (3615)	—	—	—	References 21^th^, P 66
8	P_2_O_5_	0 (2355)	38 (3345)	75 (4425)	113 (5078)	150 (5153)	188 (4275)	—	—	References 15^th^, P 81
9	CS	0 (480)	300 (1208)	450 (14550)	600 (1733)	750 (1995)	900 (2160)	1125 (2325)	1500 (2355)	References 15^th^, P 83

Note: The data in parentheses are the yields of winter wheat corresponding to the application rates, and the numerical values have been converted to kg/hm^2^ based on original trial data in the table. CS means calcium superphosphate.

**Table 5:** Comparison of fitting results between unary unstructured fertilizer response models and unary quadratic polynomial fertilizer response models on N and P fertilizer responses of winter wheat.

No.	Nutrients	Model (4)						Model (1)					
		Model coefficient			Statistical index			Model coefficient			Statistical index		
		*A*	*c* × 10^3^	*s*_0_	*F*	*R* ^2^	*S*	*b*_0_	*b*_1_	*b*_2_	*F*	*R* ^2^	*S*
1	N	69.533	4.788	58.298	39.6**	0.964	294.4	4099.6	40.684	−0.1326	112.5**	0.987	174.7
2	N	41.601	5.185	104.04	44.9*	0.978	61.3	4363.9	13.881	−0.0688	11.5	0.920	117.6
3	N	21.740	2.181	99.585	27.2*	0.948	273.4	2149.0	16.325	−0.0288	35.1**	0.959	242.0
4	Urea	21.548	1.864	187.08	66.5**	0.978	150.5	4034.5	12.928	−0.0211	101.8**	0.986	122.1
5	N	55.004	5.301	39.774	2101.8**	0.999	31.7	2212.3	38.324	−0.1482	361.1**	0.997	76.4
6	N	28.639	3.821	139.48	279.4**	0.996	24.8	4001.0	12.052	−0.0516	170.7**	0.994	31.7
7	N	41.662	5.907	58.364	109.3**	0.991	69.7	2443.7	23.521	−0.1105	167.1**	0.994	56.4
8	P_2_O_5_	64.806	6.027	33.830	17.7*	0.922	388.8	2190.3	43.746	−0.1696	46.9**	0.969	245.1
9	CS	27.635	4.508	16.238	446.7**	0.994	56.9	464.3	22.879	−0.0683	1582.8**	0.998	30.3

The unary quadratic polynomial fertilizer response model represents the symmetric function around the maximum fertilization^8^. Although the model has a 60% success rate in practice^10,11^, there is obviously a linear positive correlation between the monomial independent variable (X) and the quadratic independent variable (X^2^) of the model, which means that the coefficient in front of X and X^2^ loses the fertilizer effect meaning. Furthermore, the binary or tertiary quadratic polynomial models derived from the unary quadratic polynomial model also have the same flaw, even resulting in serious problems such as multicollinearity^2^. As a result, more than half of the binary and tertiary fertilizer response models were non-typical models that have lost their practical value^10–12^. Model (4) of the unary non-structured fertilizer response is a non-linear model that cannot be directly converted into a linear model but has well overcome the unreasonable assumption and multicollinearity of the quadratic polynomial fertilizer response model.

According to the Taylor expansion of higher mathematics, ${{e}}^{{x}}=1+{x}+{\rm{G}}({x}),{x}\in (-\infty ,+\infty )$ and ${\rm{G}}(x)=\frac{{x}^{2}}{2!}+\cdots +\frac{{{x}}^{{\rm{n}}}}{n!}+\cdots \,$. If considering only the top two items, then model (4) could be transformed into the following mathematical expression: Y = *As*_0_ + (*A* − *As*_0_*c*)X − *Ac*X^2^. This shows the same mathematical form as model (1). Therefore, model (1) of the unary quadratic polynomial fertilizer response model can be seen as a simplified and special form of model (4) of the unary non-structured fertilizer response model. If some of the experimental fertilizer data can shift the residual amount, G(x), small enough, then both models can fit well. If the residual amount of G (x) is larger, model (1) is a poor fit due to oversimplification, but model (4) still fits well.

Therefore, the unary non-structural fertilizer response model has a wide application scope. At the same time, the model provides a basic model that can be expanded to be a binary or tertiary non-structural fertilizer response model because model (4) contains the parameter of soil nutrient supply equivalent.

### Recommended fertilization rate of the unary non-structural fertilizer response model

The analysis shows that the correlation coefficients of the maximum fertilization rate and the economic fertilization rate between model (4) and model (1) were 0.987**(n = 16) and 0.991**(n = 16), respectively. This result indicates that the recommended fertilization based on the unary non-structural fertilizer response model was successful and reliable. existing previous report^1,22^ indicates that the curve or surface of the quadratic polynomial fertilizer response model becomes fairly flat at the point near the maximum yield. This phenomenon leads to a generally higher recommended fertilization rate. The comparison with Fig. 2 shows that the maximum yield of the unary non-structural fertilizer response model was generally lower than that of the unary quadratic polynomial fertilizer response model. The results of 16 field experiments that passed tests of significance using model (1) showed the maximum and economic fertilization rates (Fig. 3) recommended by the unary non-structural fertilizer response model was only 88.9%–91.5% of the unary quadratic polynomial model on average, which indicates that the new model has overcome the problems of high recommended fertilization rates by the quadratic polynomial model.

## Materials and Methods

### Rice field experimental design and processing

Eight field experiments testing the response of paddy rice to N, P and K fertilizers were performed in the main rice production regions of Fujian province, China, from 2015 to 2016. Fertilizer treatment consisted of eight combinations of different rates of N, P, or K in grey clay soil and yellow clay soil, which are the main rice soil types in Fujian province. Based on P_2_O_5_ applied at 67.5 kg/hm^2^ and K_2_O_5_ at 120 kg/hm^2^, eight N fertilization rates were combined as 0.0, 37.5, 75.0, 112.5, 150.0, 187.5, 225.0 and 262.5 kg/hm^2^, respectively. Because of the higher soil fertility after vegetable harvest, a rice paddy experiment with different N fertilization rates was conducted in Pinghe County of Zhangzhou with N application rates of 0.0, 22.5, 45.0, 67.5, 90.0, 112.5, 135.0 and 157.5 kg/hm^2^, combined with P_2_O_5_ at 30 kg/hm^2^ and K_2_O_5_ at 75 kg/hm^2^. Based on N applied at 150 kg/hm^2^ and K_2_O at 120 kg/hm^2^, eight P_2_O_5_ fertilization rates were combined as 0.0, 22.5, 45.0, 67.5, 90.0, 112.5, 135 and 157.5 kg/hm^2^, respectively. Finally, based on N applied at 150 kg/hm^2^ and P_2_O_5_ at 67.5 kg/hm^2^, eight K_2_O fertilization rates were combined as: 0, 30, 60, 90, 120, 150, 180 and 210 kg/hm^2^, respectively.

The experimental plot size was 20 m^2^ with three replications and a random block design. The main local varieties were selected as the experimental rice varieties and are widely planted in large areas. Urea (N 46%), calcium superphosphate (P_2_O_5_ 12%), and potassium chloride (K_2_O 60%) were used as fertilizers. The fertilizers for basal dressing included all of the P_2_O_5_, 50% N and 50% K_2_O; approximately 40% of the N was top-dressed at the tillering stage and another 10% of the N and 50% of the K_2_O was top-dressed at the heading stage. At harvest, the fresh weight and drying weight of rice straw and grain in each plot were measured separately. The other field management activities followed common practice.

### Sampling and statistical procedures

Soil samples were taken before planting and tested by conventional methods^23^ as shown in Table 6. Soil pH was measured with a potentiometer, organic matter was measured using the volumetric method with potassium dichromate, available N was measured using the alkaline hydrolysis diffusion method, available P was measured using 0.5 mol/L sodium bicarbonate lixiviation-Mo-Sb anti-spectrophotometer and available K was measured using 1 mol/L ammonium acetate lixiviation- flame photometer. At harvest, the fresh weight and drying weight of rice straw and grain from each plot and each treatment were measured. With conventional methods^23^, H_2_SO_4_-H_2_O_2_ digestion and total plant N, P and K were measured using distillation, vanadate-molybdate-yellow colourimetry and flame photometry, respectively.

**Table 6:** Physical and chemical properties of observed rice soils with 8 fertilization levels of N, P and K fertilizers.

Nutrients	No.	Sites	Soil types	Testing results				
				pH	OM (g/kg)	AN(mg/kg)	Olsen-P (mg/kg)	AK (mg/kg)
N	1	Datian county	GYCS	5.67	29.16	124.6	53.3	90.0
	2	Xiuyu District	YCS	5.56	34.94	157.6	40.0	41.5
	3	Pinghe county	GCS	5.66	22.44	196.0	89.0	146.5
P	4	Nan’an city	GYCS	5.87	25.50	97.6	13.0	97.9
	5	Yongchun county	GCS	5.80	27.74	180.1	15.0	97.6
	6	Datian county	GYCS	6.20	38.89	165.1	19.8	100.5
K	7	Datian county	GYCS	5.92	35.77	135.1	30.4	73.9
	8	Yongchun county	GCS	5.90	29.51	183.1	16.0	118.5

Notes: OM is organic matter in soil, AN is alkali-hydrolysable nitrogen, AK is available K in soil, GYCS is grey yellow clay soil, YCS is yellow clay soil, and GCS is grey clay soil.

Parameter estimation for the quadratic polynomial fertilizer response model was conducted using the method of ordinary least squares. Non-linear least squares methods were applied to estimate parameters of the non-linear model. We assumed the nonlinear model was Y = *f* (X, *β*). To solve the estimated value of parameter *β*, the least squares equation is min ${Q}({\beta })={\sum }_{i=1}^{n}{({\rm{Y}}i-{f}({\rm{X}}{i},{\beta }))}^{2}$. Its solution, $\hat{\beta }$ is a parameter to estimate. The statistical analyses and parameter estimations of the fertilizer response model were conducted using MathWorks MATLAB version 2015b^24^ (https://cn.mathworks.com/programs/trials/trial_request.html). We used the “regress” functions for regression analysis and statistical tests of the unary quadratic polynomial fertilizer response model, and used the “nlinfit” functions for parameter estimation and statistical test of the non-linear model. The graphs in the paper were created using MATLAB language programming.

### Other rice paddy field trials of N, P and K fertilizers

The authors have carried out ten rice field experiments on the yield response to N, P and K fertilization with 4 to 7 application rates in the past 10 years. The field trial design, sampling and laboratory analysis methods are the same as those shown in Table 6. The results are shown in Table 7.

**Table 7:** Physical and chemical properties of observed rice soils with 4 to 7 application rates of N, P and K fertilizers.

Nutrients	No.	Times	Trial sites	Levels	Soil types	Testing results (g/kg, mg/kg)				
						pH	OM	AN	Olsen-P	AK
N	9	2012	Pinghe county	6	GCS	5.55	19.68	196.0	35.6	144.0
	10	2009	Xiuyu District	6	YCS	4.80	12.50	128.8	21.5	39.1
	11	2007	Pinghe county	5	GCS	5.61	14.60	144.2	92.7	42.7
	12	2013	Datian county	5	YCS	6.09	33.27	123.7	82.5	113.0
P	13	2012	Datian county	6	GCS	5.57	26.17	155.07	123.0	89.0
K	14	2013	Datian county	7	GCS	5.04	35.83	162.4	44.0	114.0
	15	2012	Datian county	7	GCS	5.37	28.40	173.8	24.7	80.0
	16	2012	Pinghe county	6	GCS	5.54	29.11	223.0	42.9	130.0
	17	2007	Pinghe county	5	GCS	5.61	14.60	144.2	92.7	42.7
	18	2013	Datian county	4	YCS	6.09	33.27	123.7	82.5	113.0

Note: CK is the no fertilization treatment and NPK is the treatment with applied N, P and K fertilizers.

### Theoretical analysis of the unary quadratic polynomial fertilizer response model

The mathematical expression of a common quadratic polynomial with one variable is Y = *b*_0_ + *b*_1_X + *b*_2_X^2^. This polynomial’s differential expression is dY/dX = *b*_1_ + 2*b*_2_X. The result shows that the quantitative relationship between crop yield increase per unit nutrition and application rate is assumed to be linear in the unary quadratic polynomial fertilizer response model. Because the algebraic symbol *b*_2_ is negative, increased yield per unit nutrient is reduced linearly with increasing application rate, and the rate of descent is constant at 2*b*_2_.

Model (1) is a parabola on a two-dimensional coordinate graph. This graph can become a eudipleural graph through the appropriate coordinate transformation that is centred on the maximum application rate. The numerical value of increasing yield responses to fertilization before and after the maximum application rate is the same. This symmetrical relationship is based on mathematical theory and the calculation method, and cannot be simulated well due to biological variation and dynamic changes in soil physical and chemical properties. In other words, it ignores the buffer effect that soil has on nutrient application and further ignores the reduced negative effects of high fertilization rates. In addition, it is not well popularized and cannot be applied to a large number of new varieties with high yield potential and fertilizer tolerance.

Further analysis of the regression variables X and X^2^ of model (1) also indicated that the correlation coefficient was as high as 0.9429^2^. Because of a high linear correlation between the two regression variables, one of the regression variables could change with the change of the other variable. This leads to a variable coefficient value, which reduces the effect of fertilizer efficiency and cannot be used to accurately measure their contribution to the yield, even resulting in abnormal results that are difficult to explain. The binary and tertiary quadratic polynomial models that can be developed from the unary quadratic polynomial model also have the same defect, and may further result in serious multicollinearity problems^2^ which restrict regression modelling accuracy and the reliability of the statistical test. This is an essential reason that a large number of non-typical patterns have been found with the application of the quadratic polynomial fertilizer response model.

In conclusion, the unary quadratic polynomial fertilizer response model and the binary and tertiary quadratic polynomial models that have been developed from the unary model have the following three defects: (1) an assumption of a linear relationship between the increased crop yield rate per unit nutrition and the fertilizer application rate, (2) an assumption that the fertilizer efficiency before and after the maximum application rate is symmetric, and (3) a strong linear correlation already exists among the regression variables. These defects greatly reduce the accuracy of the models to simulate experimental field results.
